# Case Series and Review of Literature of Malignant Adrenocortical Neoplasms: Experience of Two Tertiary Centers in Saudi Arabia

**DOI:** 10.7759/cureus.15118

**Published:** 2021-05-19

**Authors:** Suliaman M Alaqeel, Abdulwahab Aljubab, Moath Alkathiri, Saud Aljadaan, Mohammad S Mallick

**Affiliations:** 1 Pediatric Surgery, Ministry of the National Guard - Health Affairs, Riyadh, SAU; 2 Postgraduate Medical Education, King Saud bin Abdulaziz University for Health Sciences, Riyadh, SAU; 3 Research Center, King Abdullah International Medical Research Center, Riyadh, SAU; 4 Pediatric Surgery, King Fahad Medical City, Riyadh, SAU; 5 Postgraduate Medical Education, Ministry of the National Guard - Health Affairs, Riyadh, SAU; 6 College of Medicine, King Saud bin Abdulaziz University for Health Sciences, Riyadh, SAU

**Keywords:** pediatrics, adrenocortical neoplasm, adrenocortical malignancy, adrenal mass, adrenal adenocarcinoma

## Abstract

Pediatric malignant adrenocortical neoplasms are among the rarest tumors encountered by pediatric surgeons and oncologists. In Saudi Arabia, only case reports exist due to the rarity of the condition. In this case series, we present five cases of malignant adrenocortical neoplasm and their clinical outcomes from two tertiary centers in Riyadh, Saudi Arabia, from 2012 to 2021. Patients ranged in age from one to eight years. We report the cases of three female and two male patients. All cases presented with hormonally active tumors. In two cases where tumors were excised with negative margins, only surgery and close follow-up were performed. In three cases, neoadjuvant and/or adjuvant therapy was required. In conclusion, for malignant adrenocortical neoplasms, the timing of diagnosis played a vital role in outcomes. Best outcome can be achieved with complete surgical excision as malignant adrenocortical neoplasms show a poor response to other treatment modalities.

## Introduction

Pediatric adrenal tumors may arise either from the cortex or medulla. Malignant adrenocortical neoplasms in pediatric patients are rare, with 0.2-0.3 cases reported per one million per year [1-3]. Females are affected more than males [1,4]. As opposed to those in adults, the majority of malignant adrenocortical neoplasms in pediatric patients are hormonally active [1,5].

In most patients, symptoms secondary to hormonal secretion from malignant adrenocortical neoplasms trigger investigation for such tumors in children [3]. The workup for detecting and staging suspected malignant adrenocortical neoplasms includes both laboratory investigations and cross-sectional imaging [5]. Laboratory examinations aim to detect hormonal abnormalities such as corticosteroid or sex-steroid-secreting tumors [5]. Cross-sectional imaging, either computerized tomography (CT) or magnetic resonance imaging (MRI), are performed to assess the local extent of the tumor and presence of metastasis [5]. To confirm the diagnosis of malignant adrenocortical neoplasms, an excisional/incisional biopsy of the tumor is needed. Malignant adrenocortical neoplasms are divided into four stages ranging from stage one, completely resected tumor, to stage four, tumor with distant metastasis [4].

We present five cases of malignant adrenocortical neoplasms from two tertiary centers in Riyadh, Saudi Arabia, from 2012 to 2021 along with their clinical behavior, management, and outcome.

## Case presentation

Case 1

A one-year-old full-term child was referred to pediatric surgery for routine circumcision. The patient had an unremarkable medical history. Upon examination, an enlarged penis was noted with some pubic hair. The rest of the examination was unremarkable. The patient was admitted and worked up for suspected precocious puberty.

**Figure 1:**
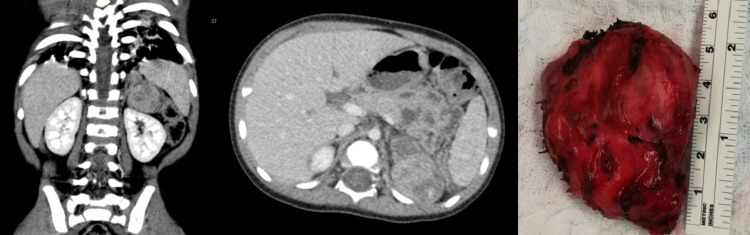
Left heterogenous suprarenal mass measuring 4.1 × 4.7 × 2.5 cm and gross image of the tumor post-resection.

Laboratory workup revealed normal electrolytes, normal cortisol, and adrenocorticotropic hormone (ACTH). Abnormal laboratory findings in the patient included elevated dehydroepiandrosterone sulfate (DHEAS), elevated insulin-like growth factor (IGF), low luteinizing hormone (LH), and low follicle-stimulating hormone (FSH). Radiological workup was performed, including hand-bone age X-ray and CT of the chest, abdomen, and pelvis. A hand-bone age scan revealed an image corresponding to a 30-month-old male. CT of the chest, abdomen, and pelvis revealed a left heterogeneous suprarenal mass measuring 4.1 × 4.7 × 2.5 cm without evidence of invasion or locoregional/distant metastasis (Figure 1).

Based on laboratory and radiological findings, the patient was elected for excision of the left adrenal mass. Through a left transverse supra-umbilical incision, a complete gross excision was achieved without spillage or rupture of the tumor. The patient’s postoperative course was unremarkable. Pathology results showed an adrenocortical neoplasm with negative margins and without clear pathological distinction whether it was an adenoma or adenocarcinoma. Two months postsurgical excision, abnormal hormonal levels noted preoperatively had normalized and there was no evidence of relapse.

Case 2

**Figure 2:**
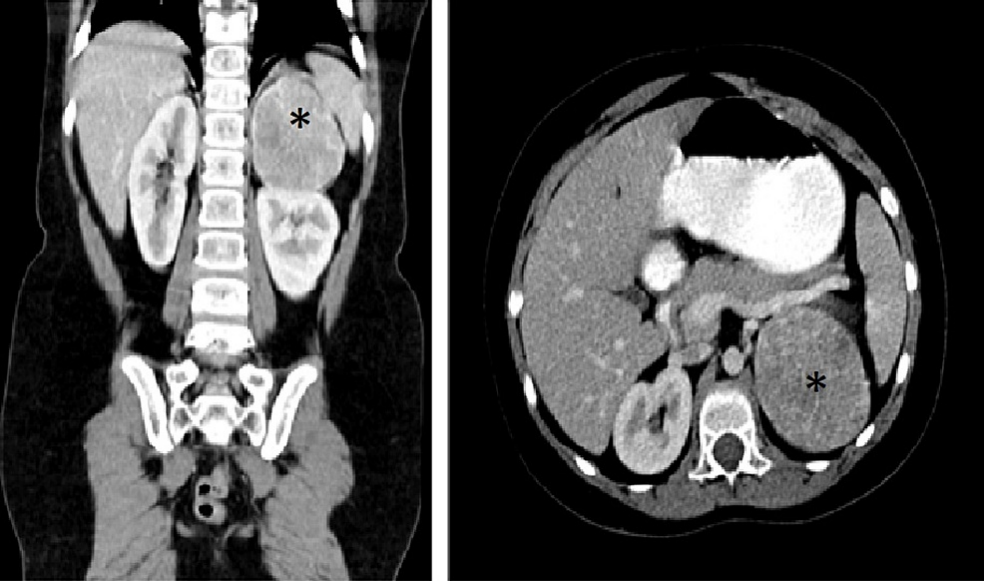
CT of the chest, abdomen, and pelvis detected a 5.5 × 6.4 × 5.3 cm left adrenal mass. CT: computed tomography

A five-year-old female presented to the clinic with a five-month history of pubic hair and increased weight. Her vital signs revealed elevated blood pressure, and her body mass index (BMI) was in the 97th percentile. Upon examination, the patient had a Cushingoid face and pubic hair. The rest of the examination, including the chest and abdomen, was unremarkable, and no palpable masses were appreciated. Laboratory workup showed low DHEAS and high cortisol levels. Other labs, including ACTH, aldosterone, and electrolytes, were within the normal range. Further investigation through CT scan of the abdomen, pelvis, and chest detected a 5.5 × 6.4 × 5.3 cm left adrenal mass (Figure 2).

The patient was admitted for left subcostal incision with complete gross excision of the left adrenal mass. Pathology tissue reports showed malignant adrenocortical neoplasm that was negative for *P53*. The patient is asymptomatic two years postexcision and shows no signs of recurrence. However, she had persistent contralateral adrenal suppression requiring steroid therapy.

Case 3

**Figure 3:**
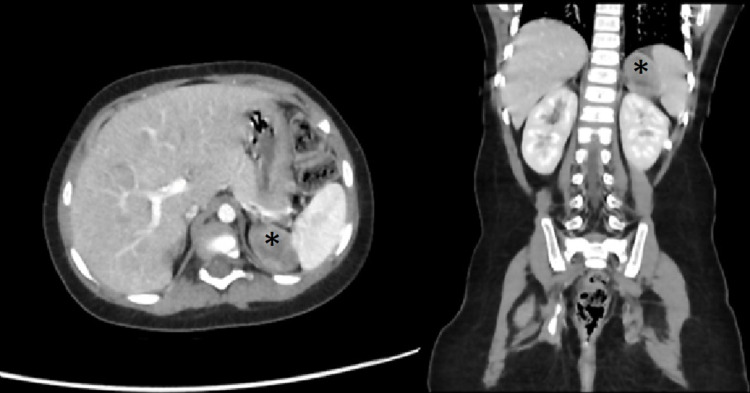
CT of the chest, abdomen, and pelvis detected a left suprarenal oval-shaped mass measuring 3.6 × 3.3 × 2.3 cm. CT: computed tomography

A one-and-a-half-year-old female presented with a three-month history of facial acne and pubic hair. The patient had a positive family history of leukemia and ovarian tumor. Her vital signs showed normal blood pressure. Physical examination revealed a Cushingoid face and pubic hair. No palpable masses or other signs of hyperandrogenism were appreciated. Labs revealed high levels of testosterone and cortisol. Other labs, including DHEAS and electrolytes, were within the normal range. Radiological workup through CT scan of the abdomen, pelvis, and chest detected a left suprarenal oval-shaped mass measuring 3.6 × 3.3 × 2.3 cm (Figure 3).

The patient was admitted for left subcostal incision and complete gross resection of the left adrenal tumor with the capsule of the tumor opened and no spillage. The pathology report showed a left malignant adrenocortical neoplasm with microscopic residual and positive *P53* gene. Due to microscopic residual, the patient was considered as stage three. The patient received postoperative chemotherapy as follows: four cycles of cisplatin, doxorubicin, etoposide, two cycles of carboplatin, doxorubicin, etoposide, and eight cycles of mitotane. The patient is now 42 months off chemotherapy with no sign of disease recurrence.

Case 4

**Figure 4:**
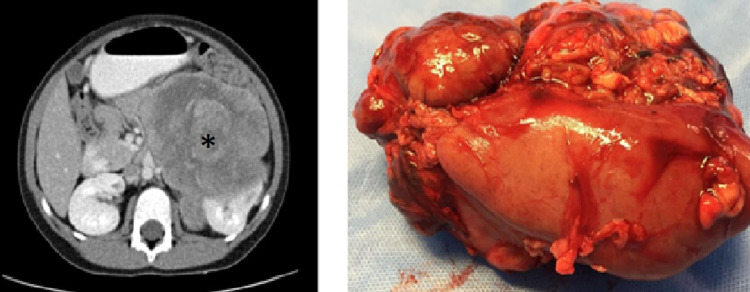
Left heterogeneous soft tissue lesion measuring 10 × 9 × 11 cm. Gross image showing the tumor invading the kidney.

A three-year-old male presented with facial acne and pubic hair. The patient’s physical examination revealed elevated blood pressure, facial acne, pubic hair, and genital enlargement. Additionally, a palpable left upper quadrant firm mass was found on abdominal examination. Laboratory investigation showed elevated cortisol, low ACTH, and elevated DHEAS and testosterone. Serum electrolytes, renin, and aldosterone were within normal limits. CT of the chest, abdomen, and pelvis was performed and showed a left heterogeneous soft tissue lesion measuring 10 × 9 × 11 cm with displacement of the splenic vessels, celiac trunk, and superior mesenteric vessels. There was no evidence of vascular encasement or distant metastasis (Figure 4).

The patient was taken for surgical excision of the mass to achieve local control through a left transverse supraumbilical incision. Intraoperative findings showed a left suprarenal mass with local invasion inferiorly to the kidney and posteriorly to the posterior abdominal wall muscles. An upfront left radical nephrectomy with adrenal mass excision was performed, including posterior abdominal wall tumor invasion. Gross macroscopic excision was done. Pathological examination showed malignant adrenocortical neoplasm with a positive margin at the area of the posterior abdominal wall invasion site. Postoperatively, the patient received oral mitotane and four cycles of chemotherapy (cisplatin, doxorubicin, and etoposide).

After receiving four cycles of chemotherapy and mitotane, the patient could not tolerate continued chemotherapy due to renal toxicity. Reassessment with CT of the chest, abdomen, and pelvis was performed three months postoperatively and chemotherapy and showed a 3.9 × 3 × 2 cm nodule in the left suprarenal space representing relapse. The patient underwent a second-look surgery through the same previous incision for resection of the relapsed mass, which was also achieved with sampling of the regional lymph nodes. Pathological examination showed viable adrenocortical adenocarcinoma with no chemotherapy changes and a positive regional lymph node. Postoperatively, oral mitotane was continued, and chemotherapy was stopped due to patient intolerance. Reassessment with CT of the chest, abdomen, and pelvis was performed one month postoperatively and showed a relapsing tumor that was nonresectable due to involvement of splenic vessels. An attempt to locally control the tumor with local radiotherapy was made, and oral mitotane systemic therapy showed a minimal response. Three months later, reassessment with CT of the chest, abdomen, and pelvis was performed, which showed two new nodules in the right 11th rib along with the tumor in the left suprarenal space with some treatment response and a decrease in size. Resection of the two nodules in the 11th rib was performed, along with continuation of local radiotherapy in the primary tumor bed, and oral mitotane was given.

Despite multimodal therapy, the disease continued to progress. CT of the chest, abdomen, and pelvis showed progressing bilateral retroperitoneal heterogeneous lesions, pelvic lesions, multiple peritoneal lesions, and sub-centimetric sub-pleural nodules on the left hemithorax. Due to poor response to the multimodal therapy approach, the patient was elected for palliative care to allow a natural death.

Case 5

**Figure 5:**
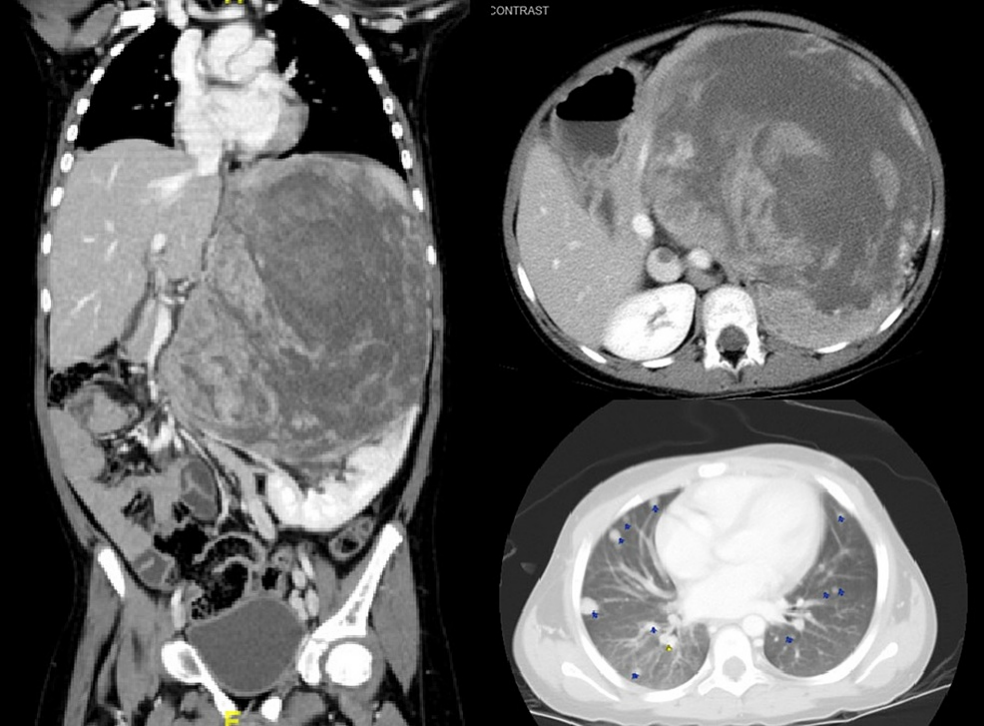
Left heterogenous suprarenal mass crossing the midline and measuring 20 × 16 × 14 cm with at least 40 lung nodules bilaterally representing metastasis.

An eight-year-old female presented to the emergency department with seizures and elevated blood pressure. After controlling the seizures and blood pressure, the patient reported a history of changes in voice and pubic hair. Physical examination showed elevated blood pressure, facial acne, hirsutism, and clitoromegaly with a left upper quadrant palpable abdominal mass. Laboratory findings showed elevated cortisol, elevated ACTH, elevated DHEAS, and elevated testosterone. The rest of labs was unremarkable. Brain MRI and CT of the chest, abdomen, and pelvis were performed. Abnormal imaging findings included a large left heterogeneous suprarenal mass crossing the midline and measuring 20 × 16 × 14 cm with inferior vena cava thrombus along with at least 40 lung nodules bilaterally representing metastasis (Figure 5).

The patient underwent ultrasound-guided biopsy using coaxial core biopsy needles of the suprarenal mass. The pathological diagnosis was malignant adrenocortical neoplasm. The management plan was to proceed for two cycles of chemotherapy and oral mitotane and to reassess later for resectability of the primary mass. Reassessment with CT of the chest, abdomen, and pelvis showed a minimal decrease in the primary suprarenal mass with progression of metastatic nodules. Due to the progression of multiple metastatic nodules and an unresectable tumor, the patient decided to undergo palliative treatment.

## Discussion

Malignant adrenocortical neoplasms are extremely rare in children [1,3]. In addition, there has been a bimodal age distribution (less than four years and more than ten years), with female patients more likely affected than male patients [1,5,6]. Malignant adrenocortical neoplasms may arise sporadically or can be associated with certain genetic alterations. Common genetic mutations include *TP53* tumor suppressor gene and insulin-like growth factor 2 (IGF2) overexpression secondary to 11p15 chromosomal locus mutation [3,7,8]. In our case series, we present five cases, three of which occurred in female patients and two in male patients with ages ranging from one to eight years. One of the cases was positive for the *P53* gene and one was negative. In three cases, testing for the *P53* gene was not performed; however, family history was negative for malignancies (Table 1).

**Table 1:** Summary of five cases. *At presentation

Case	Age/Gender	Presentation	Tumor*	Metastasis*	Stage*	Surgery	Chemotherapy	Mitotane	Radiotherapy	Follow-up period*	Survival
1	1 year/Male	Virilization	Left, <5 cm	No	1	Excision	No	No	No	6 months	Alive. Disease-free
2	5 years/Female	Virilization, Cushing’s syndrome	Left, 5.5 × 6.4 × 5.3 cm	No	1	Excision	No	No	No	26 months	Alive. Disease-free
3	1.5 years/ Female	Virilization, Cushing’s syndrome	Left, 3.6 × 3.3 × 2.3 cm	No	3	Excision	Adjuvant	Yes	No	53 months	Alive. Disease-free
4	3 years/Male	Virilization, Cushing’s syndrome	Left, 10 × 9 × 11 cm	Locoregional lymph nodes	3	Excision	Adjuvant	Yes	Yes	24 months	Death
5	8 years/Female	Virilization, Cushing’s syndrome	Left, 20 × 16 × 14 cm	Locoregional lymph nodes, lung, vascular thrombus	4	Core-needle biopsy	Neoadjuvant	Yes	No	6 months	Death

Malignant adrenocortical neoplasms may be functional or nonfunctional [1,5]. Unlike adults, most children, especially those younger than four years, present with functional tumors that trigger investigations [1,5]. Presentations include virilization, Cushing’s syndrome, hyperaldosteronism, or mixed symptomology [9,10]. The most common presentation is mixed symptomology [1,9,10]. Symptoms secondary to hyperaldosteronism are rarely seen in children with malignant adrenocortical neoplasms [5]. We present five cases with malignant adrenocortical neoplasms, which were all hormonally active mainly for hypercortisolemia and androgen hormone-secreting tumors. Consequently, these patients presented with either virilization alone or virilization with Cushing’s syndrome. None of our cases presented with hyperaldosteronism or were asymptomatic at presentation.

In malignant adrenocortical neoplasms, treatment options include surgery, mitotane, chemotherapy, and radiotherapy [1,9]. Complete surgical resection provides the best outcome for patients with malignant adrenocortical neoplasms and is considered the mainstay of treatment [4,5,9]. Mitotane, a steroidogenesis inhibitor and cytostatic antineoplastic, has been used as a neoadjuvant/adjuvant/salvage therapy in adrenocortical carcinomas [1]. Mitotane has been shown to have a positive impact on both survival and decreasing disease recurrence [5,9,11]. Despite different treatment strategies, the prognosis for metastatic disease is poor, with a five-year survival of <20% [1,12]. Notably, malignant adrenocortical neoplasm was aggressive and responded poorly to systematic therapy in two out of five patients presented in this paper. Multiple chemotherapy regimens have been attempted, and the best protocol is yet to be determined [1,5].

The overall two-year and five-year survival rate in pediatric malignant adrenocortical neoplasms is 61% and 46%, respectively [1]. The prognosis of malignant adrenocortical neoplasms can be affected by multiple factors. Patients ages four years or younger tend to have better outcomes [4,13,14]. Additionally, tumors smaller than 5 cm and weighing less than 100 g with negative resection margins are associated with better survival [1,4,14]. Finally, the extent of the disease to locoregional structures and the presence of metastasis worsen the outcome of patients with adrenocortical tumors [4]. In our review, the survival rate was 60% with a median follow-up of 24 months (Table 1).

## Conclusions

Malignant adrenocortical neoplasms are rare in children, and our review is the largest known series reported from Saudi Arabia. Based on the cases we reviewed, the timing of diagnosis plays a vital role in prognosis as complete surgical excision provides the best outcome for adrenocortical tumors. Late diagnosis with metastatic lesions predicts an unfavorable outcome as adrenocortical tumors show a poor response to both chemotherapy and radiotherapy. Further studies are needed in our region with a larger patient population to identify predictors of outcome in our region.
